# The Development of Macrophage-Mediated Cell Therapy to Improve Skeletal Muscle Function after Injury

**DOI:** 10.1371/journal.pone.0145550

**Published:** 2015-12-30

**Authors:** Viktoriya Rybalko, Pei-Ling Hsieh, Melissa Merscham-Banda, Laura J. Suggs, Roger P. Farrar

**Affiliations:** 1 Department of Kinesiology, The University of Texas at Austin, 1 University Station D3700, Austin, TX 78712, United States of America; 2 Biomedical Engineering, Cockrell School of Engineering, The University of Texas at Austin, 107 W. Dean Keaton, Austin, TX 78712, United States of America; University of Rome La Sapienza, ITALY

## Abstract

Skeletal muscle regeneration following acute injury is a multi-step process involving complex changes in tissue microenvironment. Macrophages (MPs) are one of the key cell types involved in orchestration and modulation of the repair process. Multiple studies highlight the essential role of MPs in the control of the myogenic program and inflammatory response during skeletal muscle regeneration. A variety of MP phenotypes have been identified and characterized *in vitro* as well as *in vivo*. As such, MPs hold great promise for cell-based therapies in the field of regenerative medicine. In this study we used bone-marrow derived *in vitro* LPS/IFN-y-induced M1 MPs to enhance functional muscle recovery after tourniquet-induced ischemia/reperfusion injury (TK-I/R). We detected a 15% improvement in specific tension and force normalized to mass after M1 (LPS/IFN-γ) MP transplantation 24 hours post-reperfusion. Interestingly, we found that M0 bone marrow-derived unpolarized MPs significantly impaired muscle function highlighting the complexity of temporally coordinated skeletal muscle regenerative program. Furthermore, we show that delivery of M1 (LPS/IFN-γ) MPs early in regeneration accelerates myofiber repair, decreases fibrotic tissue deposition and increases whole muscle IGF-I expression.

## Introduction

Skeletal muscle regeneration is a multi-step process involving multiple cell types and complex regulatory interactions [1, 2]. Inflammatory cascades following acute injury need to be properly managed in order for the repair to take place. Unresolved inflammatory response may lead to persistent tissue damage by immune cells or collagen deposition [3]. The severity of the inflammatory response largely depends on the type, location and extent of injury. Tourniquet-induced ischemia-reperfusion (TK-I/R) injury affects large areas of the skeletal muscle causing extensive tissue necrosis, prolonged inflammatory response and delayed recovery of muscle force and function [4, 5]. Tourniquets (TK) are used in clinic for plastic, reconstructive and orthopaedic surgeries to create bloodless surgery fields. Although complications are rare in young, tourniquet application can lead to serious complications and loss of muscle function in elderly and those with vascular pathologies [6]. The upper limit of tourniquet application in clinic is 2 hours. Irreversible muscle damage may occur when skeletal muscle is subjected to ischemia beyond 3-hour timepoint [7].

Recovery from TK-I/R injury involves extensive rehabilitation and in some cases impairments/loss of muscle function. With 20,000 TKs applied daily in the field of reconstructive and orthopaedic surgery, I/R injuries to skeletal muscle present a serious clinical problem with potential loss of man-hours to the workforce due to impaired muscular function[8]. The efficacy of existing treatments is limited stimulating the need for therapeutic approaches aimed at effective restoration of skeletal muscle function [9–13].

Several MP ablation models have demonstrated the importance of this cell type in orchestrating skeletal muscle regeneration [14–20]. MPs are prevalent and, therefore, functionally dominant at multiple phases of skeletal muscle repair, including degeneration phase, inflammation and regeneration [21].

During muscle degeneration MPs are required for necrotic muscle debris clearance and uptake of apoptotic neutrophils to clear the way for new tissue growth [3, 22]. The necrotic debris contains endogenous danger signals, such as high-mobility group box1 protein (HMGB-1), DNA and components of extracellular matrix. Binding of these signals to toll-like receptors (TLRs), specifically TLR-4 in case of HMGB-1 which is released after I/R injuries, results in classical MP activation (M1-like) [23, 24]. During classical activation of MPs, multiple inflammatory mediators are released including nitric oxide (NO), tumor necrosis factor (TNF), interleukins -1, -6, and -23 [23]. M1 activation of MPs during inflammatory phase of muscle repair coincides with myogenic activation, namely proliferation and migration of satellite cells and myoblasts [21, 25–27]. Satellite cells were shown to recruit monocytes/MPs using five different chemotactic systems and use them as support cells to reduce apoptosis [28]. The release of urokinase-type plasminogen from MPs aids in degradation of extracellular matrix proteins and proteolytic activation of hepatocyte growth factor [29, 30]. In TK-I/R injury model MPs undergo phenotypic switch as early as 3 days post-reperfusion from M1-like (F4/80^+^Ly-6C^hi^) inflammatory to M2-like (F4/80^+^Ly-6C^low^) pro-regenerative cells [31]. M2 MPs participate in final stages of skeletal muscle repair via release of anti-inflammatory mediators such as TGF-β and IL-10 and pro-regenerative factors like SLP-I, IGF-I and VEGF [3, 17, 32, 33]. M2 MPs stimulate myogenic differentiation and promote myocyte fusion [26]. Depletion of M2 MPs diminishes muscle repair and growth [34] while interference with M1-to-M2 MP transition has detrimental consequences on muscle regeneration [35].

Classically activated M1 and alternatively activated M2 MPs can be easily raised *in vitro* representing two extremes of MP activation. Due to incredible MP plasticity, mixed MP phenotypes are commonly identified *in vivo*, which makes sub-typing and classification of macrophages very complex [25, 36]. Nevertheless, *in vitro* polarized MPs have beneficial effects on muscle repair when transplanted into injured skeletal muscle [37–40]

In this study, we delivered bone marrow-derived LPS/IFNγ-polarized M1 MPs 24 hours after TK-I/R injury into damaged gastrocnemius muscle and showed significantly improved muscle function recovery 14 days post-I/R. Histological evaluation of muscle tissue showed changes in myofiber cross-sectional area and decreased collagen accumulation after M1 (LPS/IFN-γ) MP delivery. Evan’s blue dye injection experiments confirmed lower level of myofiber damage after M1 (LPS/IFN-γ) MP treatment. In the course of muscle regeneration, transplanted MPs underwent phenotypic transition evident by surface upregulation of CD206 marker. Whole muscle IGF-I expression was increased after M1 (LPS/IFN-γ) transplantation. Overall, early delivery of M1 (LPS/IFN-γ) MPs into I/R injured muscle early in the repair process aids in muscle regeneration after TK-I/R injury.

## Materials and Methods

### Animals

Male and female C57BL/6 mice (3–6 mo; Jackson Laboratories) were used for this study. Animals were housed with *ad libitum* access to food and water, and maintained on a 12-hour light/dark cycle. All experimental procedures were approved and conducted in accordance with the guidelines set by The University of Texas at Austin IACUC (Protocol # AUP-2014-00259).

### Tourniquet application

As previously described, mice were anesthetized with 2% isoflurane gas, and a single, randomly selected hind limb was elevated. A pneumatic tourniquet (TK) (D.E. Hokanson, Inc.) was wrapped snuggly against the proximal portion of the limb and inflated to 250 mm Hg by the Portable Tourniquet System (Delfi Medical Innovations Inc.) to ensure complete occlusion of blood flow to the limb. Body temperature was maintained at 37±1°C with the use of a heat lamp during this procedure. After 2 hours, the pneumatic TK was removed, and the mouse was returned to its cage for recovery. Muscles from the uninjured contralateral limb served as internal controls. Animals were euthanized between 10am-2pm at 14 days after TK-induced injury via isoflurane inhalation followed by exsanguination.

### Bone marrow isolation and macrophage polarization

Hind limb bones from donor mice were isolated and cleaned from associated muscle and connective tissue. Bones were sprayed with ethanol and rinsed 4 times in phosphate-buffered saline, pH 7.4 (PBS). Bone marrow (BM) from femurs and tibias was flushed out using PBS-filled syringe/27G needle, filtered and re-suspended in Dulbecco’s modified eagle’s medium (DMEM) supplemented with heat-inactivated 10% fetal bovine serum, 1% antibiotic/antimycotic and 10 ng/ml macrophage colony-stimulating factor (M-CSF) at 1.5-2x10^6^ cells/ml in 6-well plates. Cells were cultured in M-CSF containing media for 5 days. For polarization, bone marrow-derived MPs were stimulated with LPS and IFN-γ at 10ng/ml in 10% FCS-DMEM on day 7 of cell culture for 42h. Unpolarized MPs were designated as M0 cells. M2 macrophages were induced after the treatment with 10 ng/ml IL-4 and IL-13 cytokines (Invitrogen) for 42h.

### Cell preparation for intramuscular injection

BM-derived MPs were treated with trypsin for 2 min at 37°C to lift cells off the tissue culture plates. Cells were washed 3 times with PBS (pH 7.4), counted using hemocytometer and injected into gastrocnemius (GAS) muscles.of mice at 2x10^6^ cells/muscle in 60–100μl of PBS 24h after TK-I/R injury. GAS muscle was chosen as a target muscle for this study due to its relatively large size, superficial location, long tendon for conducting force measurements and its critical role in locomotion.

### Evan’s Blue Dye injection and tissue harvest

Evan’s blue dye (EBD) injection was performed as previously described by Hamer et al [41]. Briefly, 1% EBD solution was sterile-filtered and injected intraperitoneally at 1% volume relative to body mass 24h prior to muscle isolation at 4, 5, 6 or 7 days post-reperfusion. Gastrocnemius muscles were placed in OCT mounting medium and frozen in liquid nitrogen-cooled 2-methyl-butane for histological analysis.

### PKH 2.6 labeling and flow cytometry

BM-derived MPs were labeled using PKH26 red fluorescent cell linker kit according to manufacturer’s instructions with minor modifications (Sigma). Cells were extensively washed prior to intramuscular (i.m.) delivery into TK-injured muscles. For flow cytometric analysis, muscle cells were isolated as previously described [31]. Cell viability was confirmed using Trypan blue. Prior to staining, cells were blocked in 1% BSA/PBS, pH7.2 with addition of Fc-block (#14-0161-82, eBioscience). Cell preparations were stained with anti-CD45-APC.Cy7 (#103116, Biolegend), anti-CD11b-PE (#557397, BD Biosciences), anti-F480-APC (#123116, Biolegend), anti-CD206-Alexa 647 (#141712, Biolegend), anti-Ly-6C-FITC (#128006, Biolegend) and anti-F4/80 APC.Cy7 (#123117, Biolegend) antibodies along with recommended isotype controls. Data was analyzed using FlowJo software. Gating strategy was determined based on isotype control staining.

### Histology

Frozen, OCT-embedded muscle samples were sectioned on a cryostat (Leica CM1900; Leica Microsystems Inc.; Buffalo Grove, IL). Hematoxylin & eosin (H&E) s and Masson’s trichrome (Polyscience, Warrington, PA, USA) staining were performed as previously described [42], and slides were observed with a light microscope (Nikon Diaphot, Nikon Corp.; Tokyo, Japan) with the 20X objective lens. Images were taken using a mounted digital camera (Optronix Microfire; Optronix; Goleta, CA). Myofiber cross-sectional area (CSA) and collagen staining were measured using ImageJ software. EBD containing sections were counterstained with DAPI (1:1000; Molecular Probe, OR, USA, D1306). Anti-mouse CD31 antibody (1:25; BD Pharmingen, San Jose CA) and anti-mouse CD45 (1:10; BD Pharmingen, San Jose CA) were used to identify endothelial cells and immune cells respectively. Anti-rat secondary antibody (1: 200) Vectastain ABC Kit (Vector Laboratories, Irvine, CA, USA) and DAB substrate (Thermo Scientific, Rockford, IL, USA) were used as detecting agents according to manufacturer’s instructions.

### Real-time PCR

RNA was extracted from frozen gastrocnemius muscles (n = 3) using Trizol Reagent (Invitrogen) or Direct-zol RNA Mini-Prep Kit (Zymo Research), treated with RNase-free DNase I (Ambion), and reverse transcribed using SuperScript III Kit (Invitrogen) according to manufacturers’ instructions. Resulting cDNA was subjected to real time PCR analysis using Bio-Rad iCycler IQ5 after addition of validated primers (Table 1) purchased from RealTimePrimers.com, and SYBR-green Master Mix (Bio-Rad). Relative gene expression was determined using the ΔΔCt method.

**Table 1:** Real-Time PCR Primers.

Gene	Forward (5’-3’)	Reverse (5’-3’)
*Actb*	AAGAGCTATGAGCTGCCTGA	TACGGATGTCAACGTCACAC
*Arg1*	GTGAAGAACCCACGGTCTGT	CTGGTTGTCAGGGGAGTGTT
*Nos2*	TGACGGCAAACATGACTTCAG	GCCATCGGGCATCTGGTA
*Tnfa*	CCCACTCTGACCCCTTTACT	TTTGAGTCCTTGATGGTGGT
*Il1b*	CCCAACTGGTACATCAGCAC	TCTGCTCATTCACGAAAAGG
*Pgk1*	GCAGATTGTTTGGAATGGTC	TGCTCACATGGCTGACTTTA
*Igf1*	CTGGTGGATGCTCTTCAGTT	GCTGCTTTTGTAGGCTTCAG
*Mrc1*	CTCGTGGATCTCCGTGACAC	GCAAATGGAGCCGTCTGTGC
*PPARG*	GCGGTGAACCACTGATATTC	TGTGGTAAAGGGCTTGATGT

### Functional Measurements

Following 14 days of reperfusion, gastrocnemius muscles from the cell and saline-injected groups were surgically isolated from all other muscles and connective tissue, and subjected to *in situ* functional measurements. The Achilles tendon was secured to the muscle lever arm of a servomotor (model 305B, Cambridge Technologies) interfaced with a computer equipped with an A/D board (National Instruments). The muscle was stimulated to contract using an Isolated Pulse Stimulator (Model 2100; A-M Systems) with leads applied to the belly of the muscle. Muscle temperature was kept constant at 37°C with warm mineral oil and a radiant heat lamp throughout the procedure. Optimal length of the muscle was determined by measuring maximal twitch tension at a stimulation of 0.5 Hz. At optimal length, the muscle was stimulated at 150 Hz to elicit the peak tetanic tension (P_o_), and was allowed 2 minutes of rest between each contraction. Data were stored and analyzed using LabView software (National Instruments).

### Statistical Analysis

Data were analyzed using Student’s t-tests, one-way ANOVA (Tukey posthoc tests), where appropriate (α = 0.05). Values are represented as mean ± SEM.

## Results

### Polarization with LPS and IFN-γ induced highly inflammatory cells

**Fig 1:**
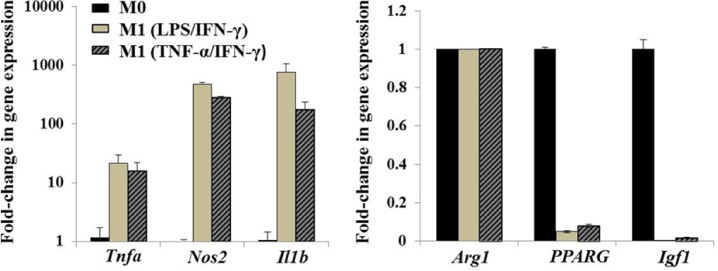
*In vitro* polarization of bone marrow-derived macrophages. BM MPs were either left untreated (M0) or treated with LPS/IFN-γ or TNF-α/IFN-γ for 42 hours to induce classical (M1) activation phenotype. Real-time PCR was performed to evaluate inflammatory (*Tnfa*, *Nos2*, *Il1b*) and anti-inflammatory (*Arg1*, *PPARG*, *Igf1*) gene expression of MPs polarized with LPS/IFN-γ or TNF-α/IFN-γ (10 ng/ml) relative to M0 MPs. β-actin was used as internal calibrator gene. Values expressed as mean ± SD.

The purpose of this experiment was to compare the activation status of differentially polarized MPs treated with lipopolysaccharide (LPS)/Type-I interferon-γ (IFN-γ) and tumor necrosis factor-α (TNF-α)/IFN-γ combinations. Classical (M1) activation was originally reported to require both IFN-γ and TNF-α, a “two signal model”. However, typical toll-like receptor ligand (TLR) ligand such as LPS can easily induce the transcription of TNF-α as well as IFN-β providing both signals and overcoming the “two signal” requirement [23, 43]. As anticipated, we saw higher inflammatory gene expression in M1 MPs treated with LPS/IFN-γ, probably due to synergistic signaling via TLR and IFN receptors (Fig 1). Relative expression of TNF-α (*Tnfa*), iNOS (*Nos2*) and IL-1β (*Il1b*) genes in M1 polarized MPs was considerably higher than that of unprimed controls (M0). As expected, there was downregulation of anti-inflammatory PPAR-γ (*PPARG*) and IGF-I (*Igf1*) gene expression in M1 polarized cells, while Arginase 1 (*Arg1*) expression was unaltered. For further studies, we used LPS/IFN-γ primed MPs, as signaling via TLR-4 receptor is important in recovery from ischemia/reperfusion injury [24].

#### In vitro polarized MPs express differential levels of CD206 surface receptor

**Fig 2:**
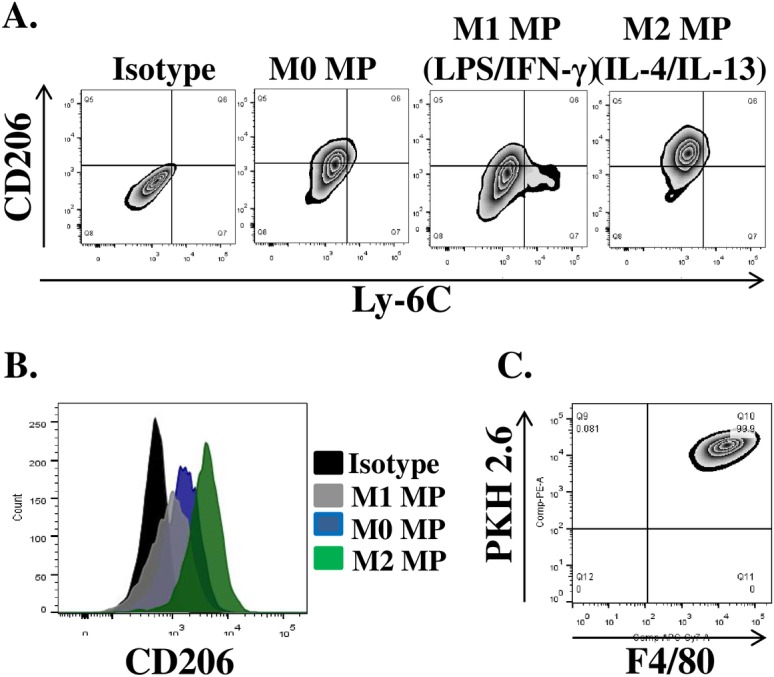
Phenotypic analysis of *in vitro* polarized BM MPs. MPs were either left untreated (M0) or stimulated with 10ng/ml LPS/IFN-γ and IL-4/IL-13for 42 hours to induce classical (M1) and alternative (M2) activation phenotypes respectively. Flow cytometry was used to evaluate the expression of CD206 and Ly-6C surface proteins. **(A)** Representative plots of surface protein after *in vitro* MP polarization, **(B)** Mean fluorescence intensity of CD206 expression on the surface of polarized MPs, **(C)** Representative plot of PKH2.6 label and F4/80expression by *in vitro* polarized macrophages prior to transplantation.

In the course of muscle regeneration MPs undergo phenotypic transition from an M1-like, inflammatory phenotype to the M2-like pro-regenerative state [32]. In general, M1 MPs are characterized by elevated expression of Ly-6C surface marker, while M2 MPs show increased expression of CD206, mannose receptor [32, 44]. We analyzed surface expression of both markers on BM-derived macrophages following in vitro polarization with LPS/IFN-γ (M1), IL-4/IL-13 (M2) compared to M0 untreated MPs (Fig 2A). Only small fraction of M1 (LPS/IFN-γ) polarized MPs (3–5%) upregulated Ly-6C expression. The mean fluorescence intensity of CD206 was different in all three treatment conditions (Fig 2B). M0 MPs showed intermediate levels of CD206 expression, M1 (LPS/IFN-γ) polarized macrophages exhibited the lowest expression levels as compared to M2 (IL-4/IL-13) polarized MPs. Unexpectedly, all three macrophage groups expressed differential levels of CD206 relative to isotype control (Fig 2B). For selected studies we additionally labelled *in vitro* polarized MPs with PKH 2.6 fluorescent membrane dye to allow for identification of transplanted MP populations after i.m. delivery (Fig 2C).

### Improved functional recovery of injured skeletal muscle after M1 MP treatment

**Fig 3:**
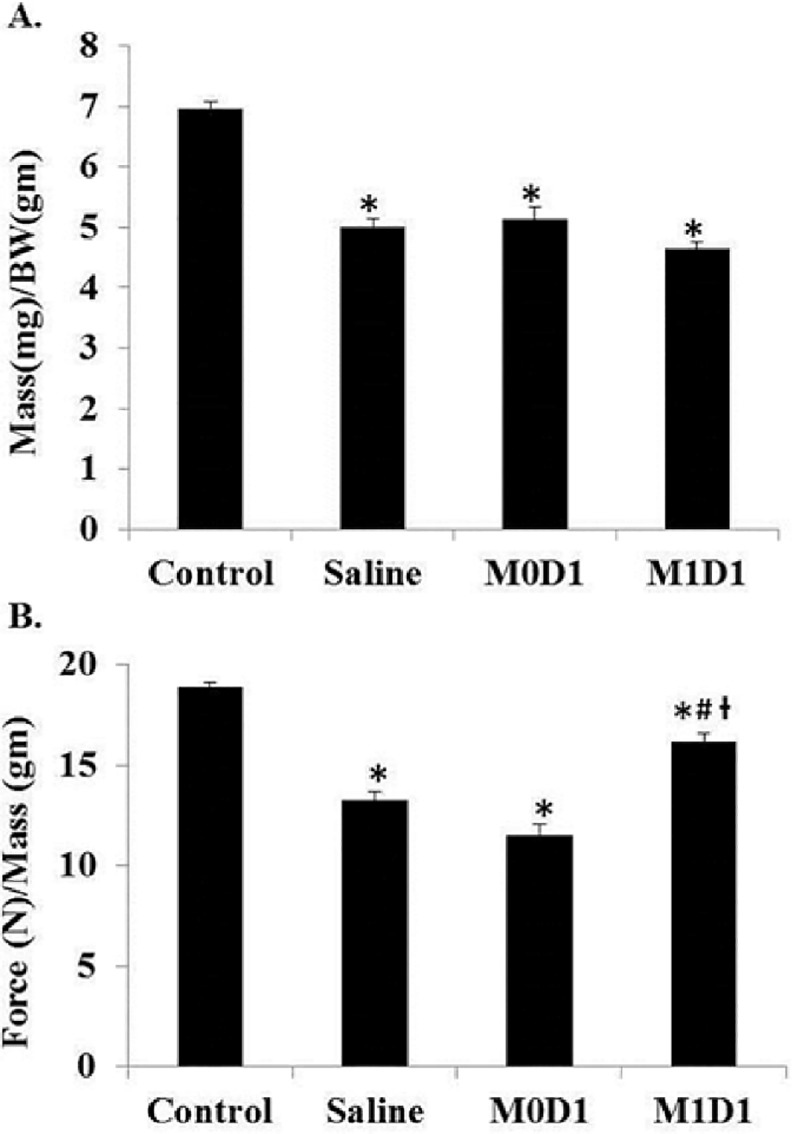
Gastrocnemius muscle mass normalized to body weight and force recovery 14 days post-reperfusion after saline injection or the delivery of 2x10^6^ *in vitro* polarized macrophages 24 h after TK-I/R injury. **(A)** GAS mass(mg)/body weight (BW)(gm); **(B)** Force (N)/GAS mass (mg). Control n = 17, Saline n = 7, M0D1 (un-polarized) MPs n = 5, M1D1 (LPS/IFN-γ polarized (42h)) MPs n = 6. Values expressed as mean ± SEM. (*) p<0.05 relative to contralateral control; (#) p<0.05 relative to saline; (ƚ) p<0.05 relative to M0D1; one-way ANOVA, Tukey-HSD post-hoc.

Functional muscle deficits are a hallmark of skeletal muscle I/R injury [5, 7]. In 2h TK-I/R models significant functional deficits in muscle function were evident at 14 days post-reperfusion [6, 45], persisting as late as 42 days post-I/R injury [46]. Despite reduced mass after TK application in all treatment groups (Fig 3A), intramuscular (IM) delivery of *in vitro* polarized M1 (LPS/IFN-γ) MPs 24h after acute TK-I/R injury significantly enhanced muscular force normalized to mass (N/gm) recovery at 14 days (85% of contralateral control) as compared to saline (70% of control), p>0.05. The infusion of M0 MPs impaired force restoration relative to saline group (60% of control) (Fig 3B). Specific tension (N/cm^2^) values confirmed these findings, except significant differences in specific tension recovery were found between saline and M0 delivery groups, with specific tension significantly reduced in the latter, p<0.05 (S1 Fig).

Taken together, our results suggest that the delivery of M1 (LPS/IFN-γ) versus M0 MPs shortly after acute muscle damage differentially impacts muscle repair processes. Elucidation of mechanisms responsible for observed effects on muscle recovery will allow for better understanding of microenvironmental components and temporal cell interactions responsible for orchestration of muscle repair.

### Improved skeletal muscle histology after M1 MP delivery

**Fig 4:**
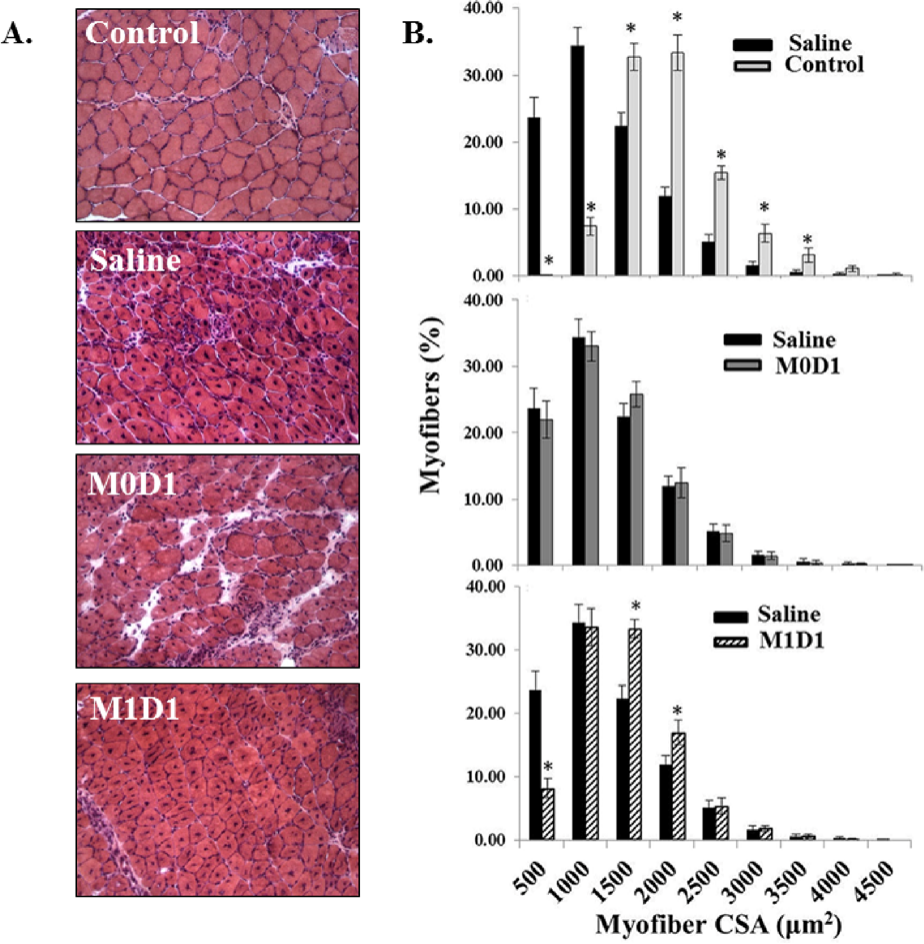
Analysis of the myofiber distribution 14 days post-reperfusion in GAS muscles treated with saline or 2x10^6^ *in vitro* polarized macrophages 24h after TK-I/R injury. **(A)** Representative H&E images of uninjured control GAS and injured, saline and MP treated GAS, at 14 days post-reperfusion; **(B) Top**: myofiber distribution in saline-treated group (black bars) relative to the contralateral uninjured control GAS(white bars); **Middle**: myofiber distribution after saline treatment (black bars) compared to M0 MP injected GAS (grey bars); **Bottom**: myofiber distribution following saline treatment (black bars) compared to M1(LPS/IFN-γ) MP treated GAS (pattern fill bars). n = 5/group; 3 fields of view/animal. Values expressed as mean ± SEM; (*) p<0.05 relative to saline; Student’s t-test.

H&E analysis of muscle tissue was performed to evaluate whether increases in muscle functional recovery after M1 (LPS/IFN-γ) MP-treatment was also associated with improvements in gross muscle morphology (Fig 4A). While all treatment groups showed an increase in central nucleation relative to control, no differences in central nucleation were observed between groups (data not shown). As anticipated, we saw significant left-hand shift towards smaller myofiber diameter in injured saline-treated muscles as compared to uninjured control (Fig 4B, top) reflecting an ongoing tissue repair after TK-I/R injury. Myofiber distribution showed no significant differences in TK-injured saline-treated and M0 MP-treated muscles (Fig 4B, middle). M1 (LPS/IFN-γ) MP-treated group showed a meaningful decrease in myofibers of diameter ≤ 500 μm^2^ and increase in larger myofibers 1000–1500 μm^2^ and 1500–2000 μm^2^ in diameter (Fig 4B, bottom).

**Fig 5:**
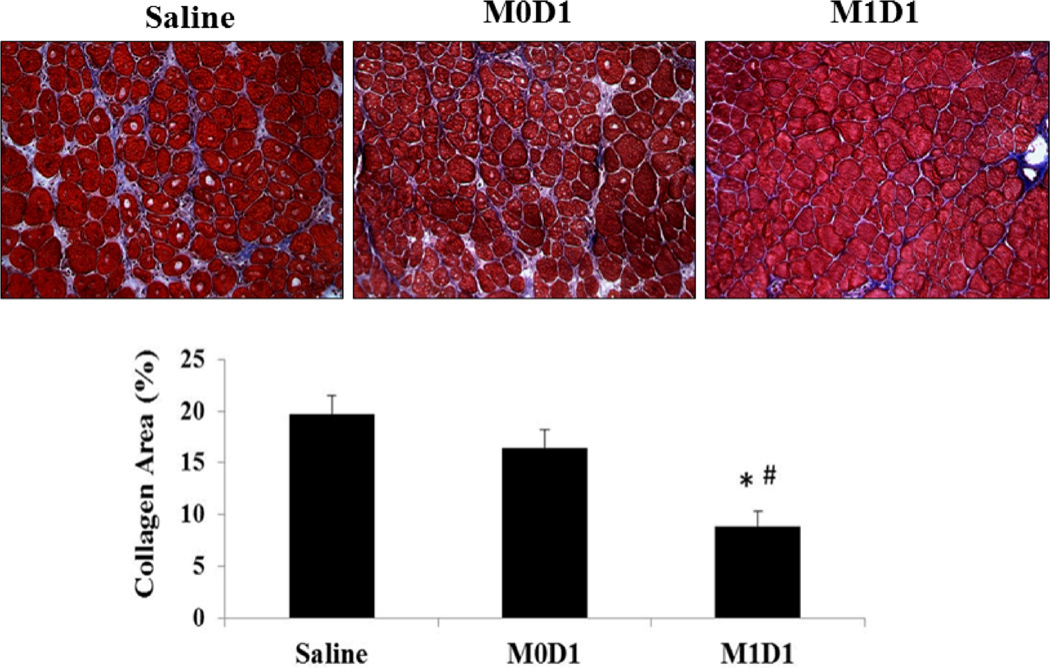
Trichrome staining for the evaluation of collagen deposition in gastrocnemius muscles 14 days post-reperfusion treated with saline or 2x10^6^ *in vitro* polarized macrophages 24h after TK-I/R injury. (*) p<0.05 compared to saline; (#) p<0.05 compared to M0D1; n = 3/group; 3 fields of view/animal; values expressed as mean ± SEM; one-way ANOVA, Tukey-HSD post-hoc.

Despite modest shifts in larger, more mature fibers, it is difficult to ascribe the enhanced muscle function solely to changes in fiber size (Fig 4B). We performed Masson’s trichrome staining in order to quantify intramuscular collagen deposition, as increased deposition of connective tissue has negative impact on contractile muscle function by decreasing myofiber occupancy. As seen in Fig 5, there is approximately 2-fold increase (16%-19% vs. 9%) in percent area occupied by collagen in saline and M0 treated groups, relative to M1 (LPS/IFN-γ) injected muscles. Overall, M1 (LPS/IFN-γ) MP delivery into TK-injured muscle 24-h post-reperfusion positively impacts histological appearance of muscle tissue as evident by the increases in myofiber diameter and reduced fibrosis.

### M1 MP treated muscles show accelerated repair

**Fig 6:**
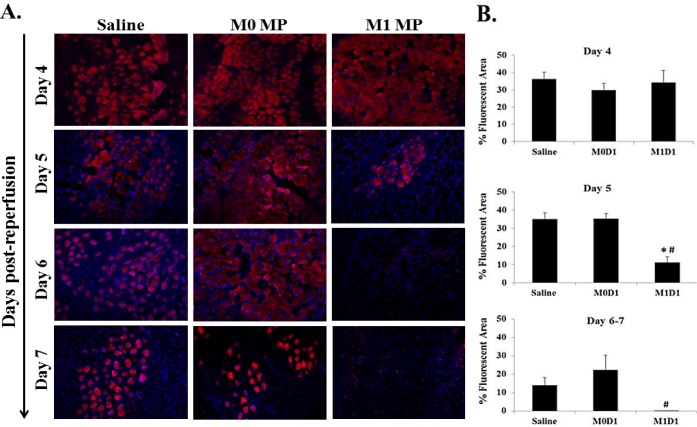
Evan’s Blue dye staining of damaged myofibers at 4, 5, 6 and 7 days post reperfusion in muscles treated with saline or 2x10^6^ M0 and M1 macrophages 24 h after TK-I/R injury. **(A)** Representative images of EBD^+^ staining (Day 4 (n = 3); Day 5 (n = 3); Day 6 (n = 1); Day 7 (n = 1)). Damaged fibers (EBD permeable) represented in red, DAPI (blue) used to counterstain cell nuclei; (B) Measurements of percent (%) fluorescent area. Days 4 and 5 post-I/R, n = 3/group, except D4-M0D1 n = 2; 3 fields of view/animal. Days 6–7, n = 2, 3 fields of view/animal. Values expressed as mean ± SEM. (*) p<0.05 compared to saline; (#) p<0.05 compared to M0D1; one-way ANOVA, Tukey-HSD post-hoc.

Debris clearance by pro-inflammatory monocytes/MPs *in vivo* has been deemed critical for tissue repair. The ablation of MPs results in persistence of necrotic tissue at the site of injury [14, 15, 18, 20]. We hypothesized that increasing numbers of M1 (LPS/IFN-γ) MPs at the site of TK-I/R injury early after acute insult could facilitate faster debris clearance by LPS/IFN-γ primed MPs. We used Evan’s blue dye (EBD) injection to label damaged muscle fibers *in vivo*. EBD^+^ muscle fibers were evident in all groups 4 days post-reperfusion without significant differences. However, starting at 5 days post-reperfusion M1 (LPS/IFN-γ) MP treated muscles showed significantly lower levels of EBD staining (Fig 6). Analysis of later time points supports this finding, with no EBD^+^ fibers in M1 (LPS/IFN-γ) treatment group evident on days 6 and 7 post-TK-I/R injury. These findings can be attributed to either increased clearance of necrotic debris by M1 (LPS/IFN-γ) MPs or accelerated M1 (LPS/IFN-γ) MP-mediated stimulation of myogenic repair processes [21, 28].

#### Transplanted MPs upregulate CD206 expression in the course of muscle repair

**Fig 7:**
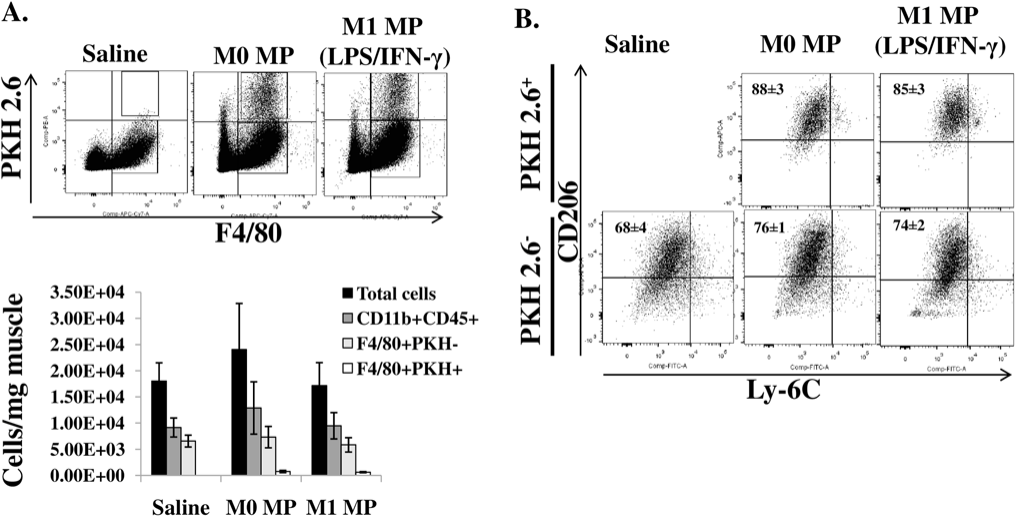
*In vivo* quantification and characterization of MP populations 5 days after TK-I/R injury. A) Flow cytometric identification and quantification of myeloid and MP cell populations in TK-injured muscle. Total myeloid cells (CD45^+^CD11b^+^), resident MPs (CD11b^+^F4/80^+^PKH2.6^-^), transplanted MPs (CD11b^+^F4/80^+^PKH2.6^+^).Values expressed as mean ±SEM, n = 3, B) Expression of CD206 and Ly-6C surface proteins on F4/80^+^MP populations in control and MP-treated muscles. Values expressed as mean ±SEM, n = 3.

MPs were shown to undergo phenotypic switching at the site of TK-I/R muscle injury [31] from an M1-like pro-inflammatory to an M2-like anti-inflammatory and pro-regenerative population[32]. Ly-6C and CD206 are two primary markers used to discriminate between pro-inflammatory (M1) MPs and anti-inflammatory (M2) MPs. We wanted to assess whether transplanted MPs were able to undergo phenotypic switching after i.m. delivery as well as to determine whether we can see any differences in the expression of Ly-6C and CD206 proteins on the surface of MP populations, both transplanted and resident, at 5 days after reperfusion injury (Fig 7). We did not see any differences in the total number of CD45^+^CD11b^+^ myeloid cells or CD11b^+^F4/80^+^ MPs among treatment groups on day 5 after TK-I/R injury (Fig 7A). Likewise, there were no differences in CD206 expression on resident F4/80^+^PKH^-^ MPs, with CD206 marker elevated on all three groups consistent with an ongoing regeneration process (Fig 7B). Both groups of transplanted MPs (M0 and M1 (LPS/IFN-γ) PKH^+^ MPs) transitioned to higher expression level of CD206 marker following *in vivo* delivery (Fig 7B). No Ly-6C expression was detected on MP populations, which is consistent with existing literature on MP phenotypes in muscle regeneration [31, 32].Although we anticipated to see higher levels of CD206 expression in the M1 (LPS/IFN-γ) treatment group on both PKH^+^ and PKH^-^ populations of MPs, no such differences were apparent at 5 days post-reperfusion injury, suggesting that if differences in MP transition exist among treatment groups, they occur between 1 and 5 days post-reperfusion.

### Increased whole-muscle IGF-I gene expression in M1 macrophage treated muscles

**Fig 8:**
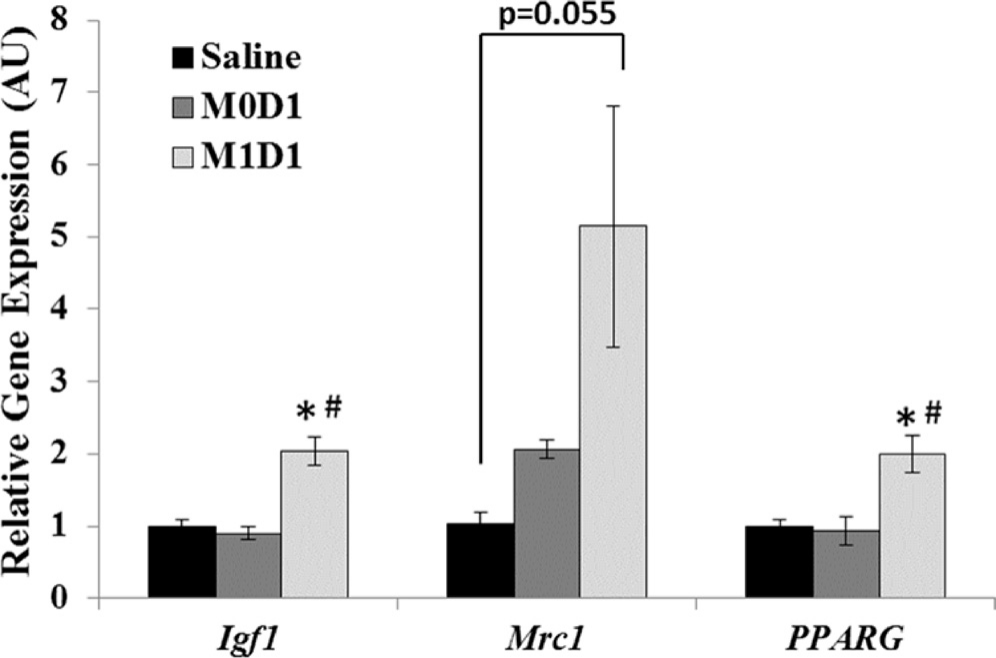
Real-Time PCR evaluation of gene expression in gastrocnemius muscles 5 days post-reperfusion treated with saline or 2x10^6^ M0 or M1 macrophages 24h after TK-I/R injury. Gene expression of IGF-I (I*gf1*), CD206 (*Mrc1*) and PPAR-γ (*PPARG*) relative to saline. Values expressed as mean transcript values ± SEM; n = 3. 3- phosphoglycerate kinase (Pgk1) was used as internal reference gene. (*) p<0.05 compared to saline; (#) p<0.05 compared to M0D1, one-way ANOVA, Tukey-HSD post-hoc.

Functional MP transition in the course of skeletal muscle repair coincides with changes in the expression of myogenic genes [21] and resolution of the inflammatory response at the tissue level [32]. The expression PPAR-γ transcription factor by MPs has been shown to aid in inflammatory resolution [32], while CD206 (*Mrc1*) mannose receptor is expressed on M2 MPs as well as murine myogenic cells [26]. IGF-I is a powerful myogenic and anti-inflammatory growth factor effecting myoblast survival, proliferation and differentiation [47, 48]. We wanted to investigate whether improved muscle recovery in M1 (LPS/IFN-γ) MP-treated muscles was associated with whole muscle changes in gene expression of PPAR-γ (*PPARG*), CD206 (*Mrc1*) and IGF-I (*Igf1*). We showed that M1 (LPS/IFN-γ) MP transplantation 1 day after TK-I/R injury results in pro-regenerative profile at 5 days, evidenced by increased expression of *PPARG* and *Igf1* over saline- and M0 MP- treated controls (Fig 8). Expression of *Mrc1* was variable, but elevated following M1 (LPS/IFN-γ) MP treatment approaching significance as p = 0.055 relative to saline treatment. This suggests that acute delivery of M1 (LPS/IFN-γ) cells into TK-I/R injured muscle may accelerate debris/apoptotic neutrophil clearance (Fig 6) [3], aid in resolution of the inflammatory response and/or promote an earlier functional MP transition from M1-to-M2-like phenotypes supported by upregulation of whole-muscle PPAR-γ, CD206 and IGF-I expression.

### Delivery of MPs differentially affects tissue revascularization and recruitment of CD45^+^ cells

**Fig 9:**
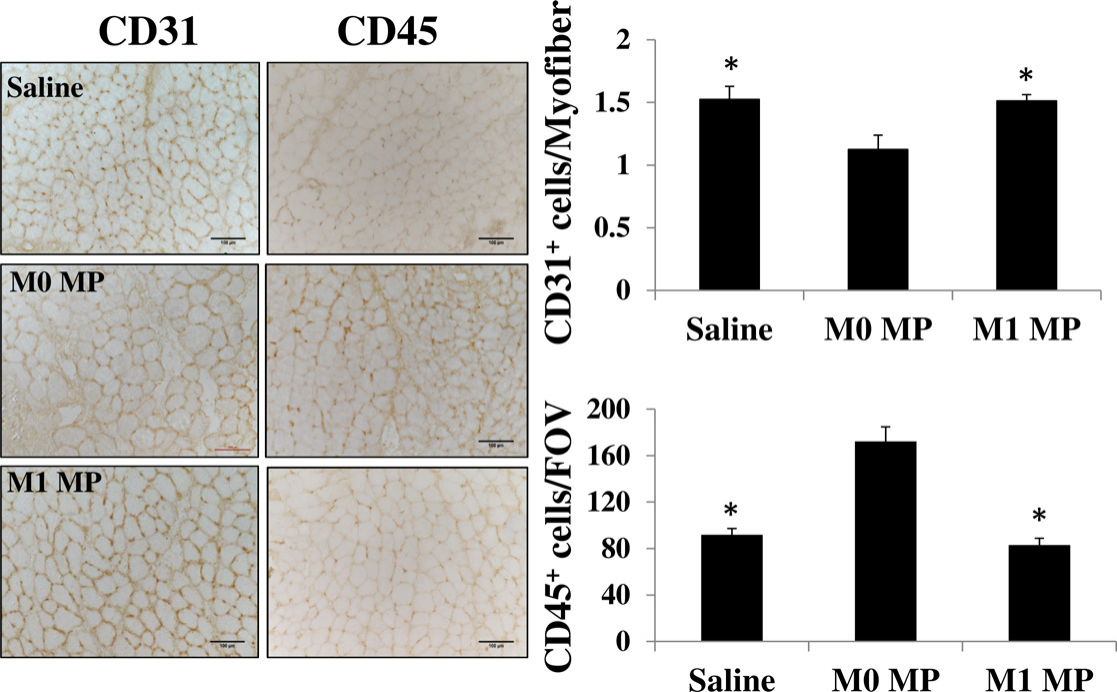
Assessment of tissue revascularization and immune cell infiltrate in saline or MP-treated TK-I/R injured muscles 14 days post-reperfusion. Representative images of D14 regenerating skeletal muscle stained with anti-CD31antibody identifying vascular endothelium and anti-CD45 staining for immune cells, scale bar 100μm. Quantification graphs of the number of CD31^+^ cell per myofiber (capillary/fiber ratio) and CD45^+^ cells per field of view (FOV). M0 group n = 3, M1 and saline groups n = 4–5, 3–5 images/muscle, values presented as mean ± SEM, (*) p<0.05 relative to M0 MP group

Microvascular cell dysfunction accounts for most of the inflammatory response associated with I/R injury and is a rate-limiting step in I/R pathogenesis [49]. Restoration of adequate microvascular supply to regenerating muscle is crucial for functional muscle recovery and performance. We, therefore, evaluated skeletal muscle revascularization in our TK-I/R injured skeletal muscles at 14 days after acute saline or MP treatment. By using CD31 specific antibodies we were able to identify endothelial cells and quantify capillary per myofiber ratio in regenerating muscles. Our results show significantly lower capillary per myofiber ratio in M0 MP treated muscles, without significant differences between saline and M1 MP treated groups (Fig 9). It is worth noting, that uninjured skeletal muscle tissue has a capillary per myofiber ratio value around 2 (data not shown). Both saline and M1 MP treated muscles showed about 80% revascularization, consistent with our histological evaluation showing an ongoing regeneration process in all three groups. Moreover, we utilized anti-CD45 staining to determine the extent of the immune cell infiltrate into regenerating muscles at 14 day after TK-I/R injury. Our data show significantly higher immune cell infiltrate into M0 MP injected muscles suggesting that skeletal muscle regeneration after M0 MP treatment may be delayed (Fig 9).

## Discussion

Increasing MP numbers at the site of injury has been associated with positive regenerative benefits. The delivery of peritoneal MPs into cutaneous wounds improves healing even in aged animals [50, 51], while additional recruitment of MPs to the site of muscle repair in the transgenic mouse model deficient in plasminogen activator inhibitor-1 improves skeletal muscle regeneration [52]. *In vitro* studies show that MPs serve as support for satellite cells [28], aid in the breakdown of extracellular matrix [29] and impact muscle progenitor cell migration and differentiation [26]. Several functional phenotypes have been characterized *in vitro*, each associated with unique function [23]. MP heterogeneity *in vivo* is a lot more complex [36, 53]. As such, polarized M1 vs. M2 MPs can be considered as two contrasting extremes with a wide range of mixed phenotypes prevalent *in vivo*.

During skeletal muscle regeneration, functionally distinct MP phenotypes have been identified [32, 35]. In our hands, after TK-I/R injury F4/80^+^Ly-6C^+^ MPs appear 24hours post-injury and gradually transition into F4/80^+^Ly-6C^-^ cells by 5 days post-reperfusion. This transition is associated with downregulation of inflammatory gene expression in MPs and upregulation of IL-10, TGF-β and IGF-I anti-inflammatory factors [31]. Increasing numbers of injury-primed, adoptively transferred Ly-6C^-^ MPs at 3 days post-reperfusion significantly enhanced functional muscle recovery [31]. The Ly-6C^+^ cells isolated from TK-I/R injury showed high inflammatory gene expression at 24-h post-reperfusion reminiscent of M1 MPs primed *in vitro*. We found that numbers of Ly-6C^+^ cells at 1 day post-reperfusion were relatively low [31], which can be explained by TK-induced vascular damage and edema. Therefore, we set out to investigate whether transplantation of *in vitro* polarized BM-derived M1 MPs 1 day post-reperfusion may have beneficial effect on muscle recovery from I/R induced damage.

We chose to use lipopolysaccharide (LPS) and IFN-γ polarization stimuli to induce M1 MPs. It has been previously suggested that LPS-stimulated macrophages may influence myogenesis at the early stages and late stages of muscle regeneration [54]. In addition, TLR-4 mediated signaling was shown to play a positive role in recovery from I/R injury [24]. In contrast, LPS is a potent inflammatory stimulant capable of aggravating I/R injury in TLR-dependent manner [55]. As such, for *in vivo* studies we chose to deliver 2x10^6^ MPs. Our rationale was based on previously published quantification of cellular infiltrate in a mouse model of TK-I/R injury [31]. Out of the total 2x10^6^ cells in a GAS muscle at 24h after TK-I/R injury, only ~35% are CD11b^+^F4/80^+^Ly-6C^hi^ MPs (~7x10^5^cells). Therefore, to ensure that our transplanted MP population would be functionally dominant, we chose to deliver 2x10^6^ *in vitro* polarized MPs to significantly enhance selected MP functional phenotype at the site of I/R injury at 24h and minimize any LPS-mediated effects. Given highly inflammatory MP profile after LPS stimulation, their numerical dominance and extensive washing prior *in vivo* delivery we were not concerned about residual LPS in our cell preparations. However, testing for LPS carry-over may become important in dose-response studies, where lower cell numbers are utilized. Similar MP cell numbers have been previously used in other injury models. For example, in a laceration injury model, delivery of 2x10^6^ MPs was shown to be effective, while lower doses had no effect [39]. We have previously treated TK-I/R injured muscle with 1.5x10^6^ adoptively transferred M2 MPs and showed improved functional muscle repair [31].

In this study we showed that *in vitro* polarized M1 (LPS/IFN-γ) MPs can beneficially affect functional muscle regeneration post-TK-I/R injury. Early delivery of polarized M1 (LPS/IFN-γ) MPs resulted in improved functional muscle recovery (Fig 3). The EBD study suggested that accelerated debris clearance and/or accelerated myofiber repair may be responsible for this outcome (Fig 6). Distinction between the two possibilities was not made in this report, but will be addressed in future studies. However, it is likely that both mechanisms take place. First, MPs have been shown to be absolutely required for necrotic tissue clearance (57). The ablation of MPs prior to tissue injury results in prolonged clearance of necrotic myofibers and tendency of muscle fat accumulation at 14 days of regeneration [20]. As such, transplantation of pro-inflammatory MPs 24 h after I/R injury may have accelerated the clearance of necrotic debris to clear way for the formation of new muscle tissue. Second, the accelerated myofiber repair can only occur via stimulation of myogenic regeneration program. MP phenotype transition was shown to coincide with onset of myogenesis [21] and precursor cell differentiation [26]. It is possible that early delivery of M1 (LPS/IFN-γ) MPs to the site of TK-I/R injury led to on site M1-to-M2 transition to hasten muscle repair. This possibility is supported by our MP characterization studies (Fig 7B) as well as gene expression data showing increased whole muscle PPAR-γ and CD206 expression, the markers of M2 MPs, as well as increased expression of the powerful pro-regenerative and anti-inflammatory factor IGF-I (Fig 8) [56]. At this point, it is unclear whether or not transplanted macrophages exhibited differential levels of IGF-I and PPAR-γ expression following phenotypic transition characterized by CD206 upregulation. Similarly we did not address changes in IGF-I expression by recruited and resident cell populations [56]. It is interesting to note that by 5 days post-reperfusion both M1 and M0 transplanted MP populations are significantly reduced suggesting that transplanted MPs either trafficked out of the injured muscle via lymphatics, or got cleared by resident MPs. In either case, there appears to be differential impact on muscle regeneration and repair. Additional studies need to address how both MP populations contribute to muscle regeneration.

Restoration of microvascular supply is essential for muscle function. Tissue revascularization in M1 MP treated muscles was similar to saline control at 14 days post TK-I/R injury. In both groups, capillary per myofiber ratio was around 80% of uninjured control value, which is consistent with ongoing regenerative process. As such, the improvements in myofiber diameter and reduced deposition of connective tissue in M1MP treated groups may contribute to functional improvements at 14 days after TK-I/R injury.

In contrast, delivery of M0 MPs 24h post-TK-I/R injury delayed regeneration as evidenced by impaired functional muscle recovery relative to saline-treated control. Although somewhat surprising given the plasticity of MPs, this result can serve as another evidence for the importance of MP activation status in controlling muscle repair. Restoration of muscle contractile function is dependent on a variety of factors, including myofiber cross-sectional area, myofiber maturity, amount of residual inflammation [57], remodeling and fibrotic tissue deposition, re-innervation and excitation-contraction coupling, just to name a few. We showed that prior to i.m. delivery M0 MPs expressed substantially lower levels of inflammatory markers (Fig 1) and intermediate levels of CD206 (Fig 2), an M2-like MP signature. It is tempting to speculate that M0 MPs may perturb the muscle microenvironment by interfering with early recruitment of inflammatory Ly-6C^+^ monocytes to the site of injury. Subsequently, changes in the recruitment kinetics of Ly-6C+ monocytes/MPs to the site of injury may in turn lead to impaired or delayed clearance of muscle debris and apoptotic neutrophils, slowing the breakdown of extracellular matrix and negatively impacting satellite cell activation. The delivery of M0 MPs may alter the timing of phenotypic and functional MP transition at the site of injury, negatively impacting myofiber repair. Although, we show that M0 MPs upregulate CD206 expression to the same extent as resident and transplanted M1 MPs at 5 days post-TK-I/R, the gene expression profile of these MPs may be different as evidenced by our whole muscle gene expression data. M0 treated muscles show no significant upregulation of PPAR-γ, CD206 and IGF-I over saline group. The increase in muscle fibrosis along with decrease in capillary per myofiber ratio and higher immune cell infiltrate at two weeks of recovery serves as an important evidence of the delayed regenerative response in M0 MP treated muscles and partially explains functional deficiencies relative to saline and M1MP treated groups. Interference with temporal transition of MPs was shown to have detrimental effects on muscle regeneration [35, 58, 59].

This report provides evidence for the importance of tissue microenvironment on controlling the outcome of the repair. Taken together, these findings show promise in the use of MPs as therapeutic modality to enhance muscle repair, but more studies need to be done on temporal regulation of MP activation status after delivery.

## Supporting Information

S1 Fig Gastrocnemius specific tension recovery 14 days post-reperfusion after saline injection or the delivery of 2x10^6^ *in vitro* polarized macrophages 24 h after TK-I/R injury. Specific tension N/cm^2^ of GAS muscle. Control n = 17, Saline n = 7, M0D1 (un-polarized) MPs n = 5, M1D1 (LPS/IFN-γ polarized (42h)) MPs n = 6. Values expressed as mean ± SEM. (*) p<0.05 relative to contralateral control; (#) p<0.05 relative to saline; (ƚ) p<0.05 relative to M0D1; one-way ANOVA, Tukey-HSD post-hoc.
(JPG)
