# Supplementary figures and images for: The Development of Macrophage-Mediated Cell Therapy to Improve Skeletal Muscle Function after Injury
Source: PLoS One. 2015 Dec 30;10(12):e0145550. doi: 10.1371/journal.pone.0145550 (PMC4696731; doi:10.1371/journal.pone.0145550)

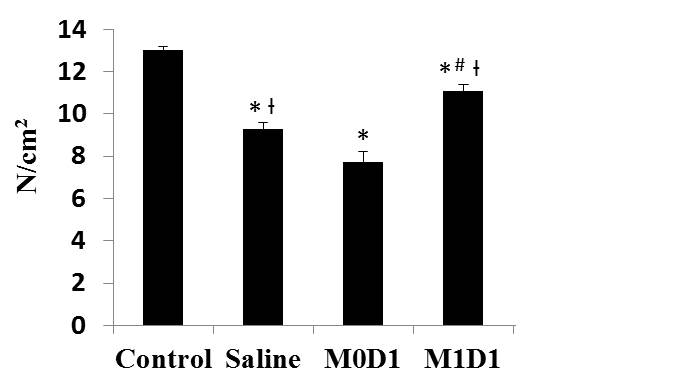

Supplement: S1 Fig — Specific tension N/cm2 of GAS muscle. Control n = 17, Saline n = 7, M0D1 (un-polarized) MPs n = 5, M1D1 (LPS/IFN-γ polarized (42h)) MPs n = 6. Values expressed as mean ± SEM. (*) p<0.05 relative to contralateral control; (#) p<0.05 relative to saline; (ƚ) p<0.05 relative to M0D1; one-way ANOVA, Tukey-HSD post-hoc. (JPG) [file pone.0145550.s001.jpg]
